# Characterising Mitochondrial Capture in an Iberian Shrew

**DOI:** 10.3390/genes13122228

**Published:** 2022-11-27

**Authors:** Henry D. Kunerth, Joaquim T. Tapisso, Raul Valente, Maria da Luz Mathias, Paulo C. Alves, Jeremy B. Searle, Rodrigo Vega, Joana Paupério

**Affiliations:** 1 Department of Ecology and Evolutionary Biology, Cornell University, Ithaca, NY 14853, USA; 2 CESAM—Centro de Estudos do Ambiente e do Mar, Departamento de Biologia Animal, Faculdade de Ciências, Universidade de Lisboa, 1749-016 Lisbon, Portugal; 3 CIBIO, Centro de Investigação em Biodiversidade e Recursos Genéticos, InBIO Laboratório Associado, Campus de Vairão, 4485-661 Vairão, Portugal; 4 BIOPOLIS Program in Genomics, Biodiversity and Land Planning, Campus de Vairão, 4485-661 Vairão, Portugal; 5 Departamento de Biologia, Faculdade de Ciências, Universidade do Porto, 4169-007 Porto, Portugal; 6 CIMAR/CIIMAR—Interdisciplinary Centre of Marine and Environmental Research, University of Porto, Avenida General Norton de Matos, S/N, 4450-208 Matosinhos, Portugal; 7 Estação Biológica de Mértola (EBM), Praça Luís de Camões, 7750-329 Mértola, Portugal; 8 Ecology Research Group, Section of Natural and Applied Sciences, School of Psychology and Life Sciences, Canterbury Christ Church University, Canterbury CT1 1QU, UK; 9 EMBL-EBI, Wellcome Trust Genome Campus, Hinxton, Cambridge CB10 1SD, UK

**Keywords:** *Sorex araneus* complex, karyotype, introgression, phylogenetics, hybridisation, Iberia

## Abstract

Mitochondrial introgression raises questions of biogeography and of the extent of reproductive isolation and natural selection. Previous phylogenetic work on the *Sorex araneus* complex revealed apparent mitonuclear discordance in Iberian shrews, indicating past hybridisation of *Sorex granarius* and the Carlit chromosomal race of *S. araneus*, enabling introgression of the *S. araneus* mitochondrial genome into *S. granarius.* To further study this, we genetically typed 61 *Sorex araneus/coronatus/granarius* from localities in Portugal, Spain, France, and Andorra at mitochondrial, autosomal, and sex-linked loci and combined our data with the previously published sequences. Our data are consistent with earlier data indicating that *S. coronatus* and *S. granarius* are the most closely related of the three species, confirming that *S. granarius* from the Central System mountain range in Spain captured the mitochondrial genome from a population of *S. araneus*. This mitochondrial capture event can be explained by invoking a biogeographical scenario whereby *S. araneus* was in contact with *S. granarius* during the Younger Dryas in central Iberia, despite the two species currently having disjunct distributions. We discuss whether selection favoured *S. granarius* with an introgressed mitochondrial genome. Our data also suggest recent hybridisation and introgression between *S. coronatus* and *S. granarius*, as well as between *S. araneus* and *S. coronatus.*

## 1. Introduction

Mitochondrial (mt)DNA sequences have long been exceptionally valuable molecular markers for systematics and specimen-based population genetics [1]. Even early in the population genetic study of mtDNA, instances of “cytonuclear discordance” [2] were demonstrated—including in small mammals [3], where populations are characterised by the nuclear genome (and morphology) of one species or subspecific taxon, but the mitochondrial genome of another species/subspecific taxon. This mitochondrial introgression, which has now been frequently observed, has been viewed as one reason to be sceptical about the use of mtDNA in phylogenetic and population genetic inference [4], but researchers are increasingly taking the view that instances of cytonuclear discordance are of interest in themselves in describing aspects of population history. In particular, there is a realisation that the integration of a foreign mitochondrial genome may often—or perhaps even typically—be advantageous, with selection favouring the process [5]. Hence, the connotations associated with the term “mitochondrial capture” (e.g., [6]) may be appropriate, and where mitochondrial introgression is observed it is worth thinking about what selective process(es) may be involved. Demographic processes and sex-biased dispersal may also be important [2]. An appreciation of what promotes mitonuclear discordance adds nuance to our understanding of the recent population history of a species.

To gain an understanding of instances of mitochondrial capture requires widespread sampling over the distribution of a species or within an area of particular geographic interest. If possible, it is good to have widespread sampling of multiple species in the same genus, to help understand whether it is the particular circumstance of the interaction of two particular species within the genus that leads to a capture event, or whether it is more pervasive than that. An exemplary case can be found in hares of the genus *Lepus* in Europe and North America. In *Lepus*, mitochondrial capture has been demonstrated in multiple species; in particular, it is the mtDNA of more cold-adapted species that has introgressed into more warm-adapted species, and these events occurred during periods of sympatry during glacial cold periods when the cold-adapted species were widespread (e.g., [7,8,9,10]).

The *S. araneus* complex consists of 10 species of red-toothed shrews with a Holarctic distribution extending from Western Europe to eastern North America, each distinguished by a different karyotype [11]. One of the species—*S. granarius*, an Iberian endemic—has a karyotype that may be ancestral to all four European species [11,12]. *S. granarius* has apparently captured the mitochondrial genome of another species in the complex—*S. araneus* s.s.—according to studies of molecular markers by Yannic et al. [13,14]. Based on mitochondrial DNA, the phylogeny of members of the *S. araneus* complex in Iberia (*araneus*, *coronatus*, *granarius* [15]) shows *S. granarius* to be most closely related to *S. araneus*, while phylogenies based on Y chromosomes or autosomal markers show *S. granarius* to be most closely related to *S. coronatus*. As well as being distinguished from closely related species by karyotype, *S. araneus* is subdivided into over 70 karyotypically distinct forms (“chromosomal races”) [11]. Of these karyotypic forms, the one found in Iberia is known as the “Carlit” chromosomal race, which has a relatively undifferentiated karyotype—one of the most similar to the “ancestral” *S. granarius* karyotype [11]. Thus, the mitonuclear discordance found in Iberian shrews indicated to Yannic et al. [14] that there had been hybridisation of *S. granarius* and the Carlit chromosomal race of *S. araneus*, enabling introgression of the *S. araneus* mitochondrial genome into *S. granarius.* A prediction from Yannic et al.’s hypothesis is that there is or was a “true” mitochondrial genome in *S. granarius*, which was displaced by the *S. araneus* mitochondrial genome over at least part of the distribution range of *S. granarius*.

We here describe what we believe to be that predicted original *S. granarius* mitochondrial genome, identified in the context of a phylogenetic analysis of mtDNA sequences of the three Iberian species of the *S. araneus* complex. While there is a large dataset of *S. araneus* mtDNA sequences already available, here we expand the number of mtDNA sequences available for *S. granarius* and *S. coronatus*, enabling a more thorough phylogenetic analysis of mtDNA variation in these species. Likewise, the new individuals were sequenced with nuclear markers to expand the analysis of mitonuclear discordance. Our results support the suggestion from Yannic et al. [14] of a mitochondrial capture event in Iberian shrews, and we consider how it might have come about. Our study also provides preliminary evidence of other hybridisation and introgression events among the three species of *Sorex* in Iberia.

## 2. Materials and Methods

### 2.1. Sampling, DNA Extraction, Sequencing, and Amalgamation of Sequences

**Figure 1:**
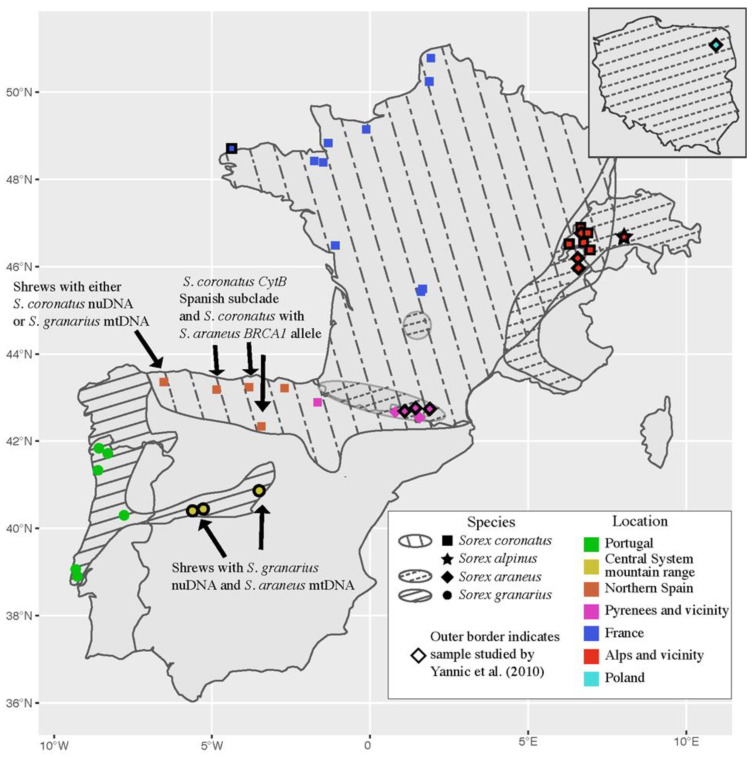
Collection localities of 61 *Sorex* shrews described here, plus 35 described by Yannic et al. [14] (see text). These localities are in southwestern Europe and Poland (inset). The specimens are classified as *S. araneus/coronatus/granarius* according to their geographic location [14] and/or nuclear DNA sequences (see text). The species distribution ranges shown on the map are derived from [14]. Interesting features of the nuclear (nu)DNA and mitochondrial (mt)DNA sequences are indicated (also see text).

DNA from 61 individuals within the *S. araneus* complex—specifically, *S. araneus* s.s., *S. coronatus*, and *S. granarius*—was used in this study. These samples were collected between 1994 and 2016 across 23 localities in Portugal, Spain, France, and Andorra (Figure 1). All tissues were stored in ethanol prior to extraction. DNA was extracted from foot, tail, and ear tissues using EasySpin Genomic DNA Tissue Kits (Citomed, Lisbon, Portugal). DNA from five genes was amplified and sequenced. The loci included a mitochondrial locus *Cytochrome B* (*CytB*), a Y-linked locus *DBY7*, an X-linked locus *Zinc Finger Protein 1* (*ZFX1*), and two autosomal genes *Breast cancer susceptibility 1* (*BRCA1*) and *Apolipoprotein B* (*ApoB*).

A total of 2970 base pairs (bp) of DNA was amplified and sequenced: 1003 bp from *CytB*, 504 bp from *DBY7*, 190 bp from *ZFX1*, 499 bp from *ApoB*, and 774 bp from *BRCA1* (Supplementary Table S1). PCRs were performed in a final volume of 5 μL containing 0.5–1.0 μL of DNA, 2 μL of MasterMix, 0.2 μL of each primer, and the remaining volume in water. Amplification was performed through 15 min of initial denaturation at 95 °C, followed by 35 cycles of 95 °C for 30 s (45 s for *CytB*), an annealing step for 30 s (60 s for *CytB*, and 45 s for *ApoB* and *BRCA1*; see annealing temperatures in Supplementary Table S1), and 72 °C for 60 s (30 s for *ZFX1*), along with a final extension step at 60 °C for 5 min. A touchdown protocol was applied to *DBY7*, with 21 cycles at 95 °C for 30 s, and decreasing annealing temperatures from 65 °C to 55 °C by 0.5 °C per cycle, with otherwise identical parameters. PCR products were purified with ExoSAP-IT^®^ PCR clean-up kits (GE Healthcare, Piscataway, NJ, USA). Sequencing reactions were performed using the BigDye^®^ Terminator v1.1 Cycle Sequencing Kit (Applied Biosystems, Carlsbad, CA, USA). Samples were sequenced on both strands on a 3130xl Genetic Analyzer sequencer (Applied Biosystems/ Hitachi, Foster City, CA, USA/Tokyo, Japan).

Sequences were assembled and edited in Sequencher v5.4.6 (Gene Codes Corp., Ann Arbor, MI, USA) and verified by eye, and then they were aligned using ClustalW [16] implemented in MEGA v11 [17]. Additional sequences from Yannic et al. [13,14] from the species *S. araneus*, *S. coronatus*, *S. granarius*, and the outgroup *Sorex alpinus* were also included and aligned. Samples were phased using the PHASE algorithm [18] implemented in DNAsp v6 [19]. All samples for each gene were phased using 100 iterations after 100 burn-in iterations.

Specifically, our data for one mitochondrial and four nuclear loci for 61 *S. araneus/coronatus/granarius* from Portugal, Spain, France, and Andorra (Supplementary Table S2) were combined with data for the same loci on 35 individuals of the same three species from Yannic et al. [14] (*BRCA1*: GU473723–GU473750, GU473766–GU473780, GU473786–GU473787; *ApoB*: GU473788–GU473816, GU473830–GU473840, GU473845–GU473846; *ZFX1*: GU473847–GU473870, GU473883–GU473893, GU473898–GU473899) supplemented with earlier published data [13] on *CytB* (EF636497–EF636516, EF636526) and *DBY7* (EF636575, EF636576, EF636580, EF636581, EF636585). The data from Yannic et al. [14] were from Spain, France, Switzerland, and Poland. The sampling points of all 96 individuals are mapped in Figure 1. We referred to Yannic et al. [14] for the species designation of their specimens. For our specimens, we did not attempt to identify them morphologically, as the species are very similar in appearance [14]. However, based on the geographical distribution of the three species provided by Yannic et al. [14], 52 of the specimens that we genotyped could be attributed unequivocally to one of the three species. Four specimens from northwestern Spain were from close to the contact area of *S. coronatus* and *S. granarius*, while five specimens were from close to the contact area of *S. araneus* and *S. coronatus* in the vicinity of the Pyrenees, at the border of Spain and France (Supplementary Table S2). In our phylogenetic analyses, we attributed these nine individuals to species on the basis of haplotypes at the markers scored.

### 2.2. Tree Building

Maximum likelihood trees were built using IQtree v1.6.12 [20] using the ModelFinder [21] algorithm to automatically identify the best-fit model for sequence evolution. Identical sequences were ignored for the purpose of tree building but presented in visualisations and subsequent analysis. Individual samples with heterozygous genotypes were included, and each ambiguous character was given equal weight when building trees.

Bootstrap support derived from IQtree is displayed on the tree (70% or more for the *CytB* tree and the concatenated nuclear gene tree, each constructed on large numbers of variable nucleotide sites; 50% or more for the individual nuclear gene trees based on few variable nucleotide sites).

For *CytB*, a haplotype network was built in R using a minimal spanning tree implemented through the R program ape 5.6–2 [22].

### 2.3. Summary Statistics

Diversity indices—specifically, haplotype counts, haplotype diversity, nucleotide diversity, and number of segregating sites—as well as the neutrality tests Tajima’s *D* [23] and Fu’s *F_S_* [24], were estimated using DNAsp v6 [19]. For each set of statistics, the data were partitioned by geography, nuclear genomic species identity, and mitochondrial species identity.

## 3. Results

**Figure 2:**
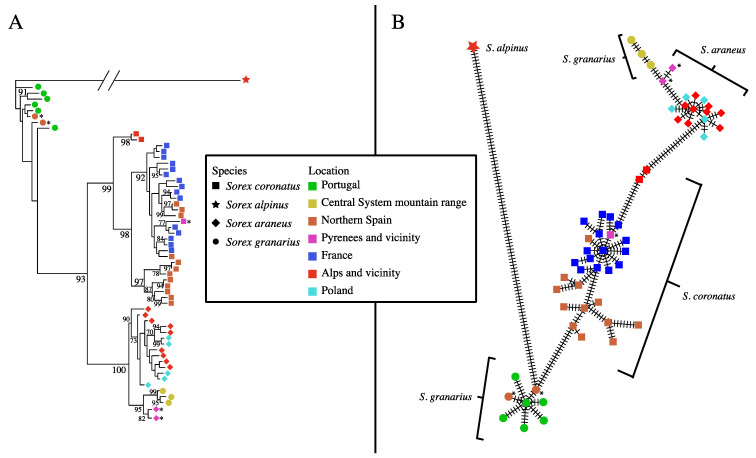
Maximum likelihood phylogenetic tree (**A**) and minimum spanning network (**B**) for mitochondrial *CytB* sequences of members of the *S. araneus* complex from southwestern Europe and Poland. Only haplotypes are shown, with geographic location and species indicated by symbols. *S. alpinus* is the outgroup. Asterisks indicate the haplotypes of individuals from geographic regions where two species occur. Their species designation is justified in the text. Bootstrap support over 1000 replicates is indicated on major internal nodes of the tree (only values of 70% or greater). In the network, bars on branches indicate mutational steps.

For *CytB*, there was a clear clade of *S. coronatus* haplotypes with subdivision into three subclades based on geography (Figure 2A). All of the French specimens were in one subclade, together with specimens from Andorra and nearby areas of Spain as far west as Burgos (Figure 1; Supplementary Table S2). A second subclade consisted of specimens from Spain, from Burgos westwards. A third subclade consisted of specimens from Switzerland. The sister clade to the *coronatus* clade consisted largely of *S. araneus*, mostly in a single subclade, but a second smaller subclade included both *S. araneus* from the Pyrenees and *S. granarius* from the Central System mountain range in Spain. Even though one of the subclades had *CytB* deriving from two species, its nucleotide diversity (0.00379, *N* = 10) was similar to that in the other subclade (0.00305, *N* = 21) and considerably lower than that of *S. araneus* overall (0.00608, *N* = 27) (Table 1). Finally, close to the outgroup branch in the tree there were eight distinct haplotypes of *S. granarius* from Portugal and northwestern Spain.

Haplotype diversity was very high (close to 1), and both Tajima’s *D* and Fu’s *Fs* were negative for all groupings in Table 1, except for some of the groupings with small sample sizes.

The haplotypes of shrews collected in regions where two species occur are highlighted in Figure 2A. The four individuals typed at *CytB* from the contact area of *S. coronatus* and *S. araneus* in the vicinity of the Pyrenees all had *CytB* haplotypes that clearly fit into one species or the other (three individuals as *S. araneus* (two haplotypes), one as *S. coronatus*), and in the appropriate subclades of those species given their geographic location. They also had nuclear genotypes consistent with their species designation (see below).

**Table 1:** Diversity statistics for the mitochondrial locus *CytB* in *S. araneus*, *S. coronatus*, or *S. granarius*, grouped according to nuclear species identity, mitochondrial grouping or geography (see Supplementary Table S2). Data are based on our new samples and those of Yannic et al. [13]. See text for further details.

Category	N Individuals	N Haplotypes	Haplotype Diversity *(Hd)*	π	Tajima’s *D*	Fu’s *Fs*
Nuclear species level						
*S. araneus*	27	16	0.940 ± 0.00073	0.00608	−0.98317	−3.873
*S. coronatus*	43	30	0.981 ± 0.00009	0.01371	−1.05151	−7.277
*S. granarius* (Portugal + Central System)	10	9	0.978 ± 0.00292	0.02512	0.73617	−0.269
mtDNA groups						
*S. araneus*	31	19	0.953 ± 0.00045	0.00787	−0.70046	−4.214
*S. araneus* (Pyrenees) + Central System *S. granarius* group	10	5	0.822 ± 0.00940	0.00379	0.33247	0.880
*S. araneus* (France, Switzerland, Poland)	21	14	0.933 ± 0.00158	0.00305	−2.06698	−7.787
*S. coronatus*	43	30	0.981 ± 0.00009	0.01371	−1.05151	−7.277
*S. granarius* (Western Iberia)	8	8	1.0 ± 0.00391	0.00716	−1.77245	−2.933
Geographic subpopulations						
*S. araneus* (Pyrenees)	6	2	0.533 ± 0.02963	0.00106	1.03194	1.723
*S. araneus* (France, Switzerland, Poland)	21	14	0.933 ± 0.00158	0.00305	−2.06698	−7.787
*S. coronatus* (Spain)	18	13	0.967 ± 0.00068	0.01260	−0.13873	−0.835
*S. coronatus* (France, Switzerland)	25	17	0.960 ± 0.00054	0.01078	−0.64136	−2.421

The two individuals typed at *CytB* from the contact area of *S. coronatus* and *S. granarius* were classified as *S. granarius*. There is no information on their nuclear genotype; however, they have *CytB* haplotypes similar to *S. granarius* from Portugal, to the south of where they were collected (Figure 1). They are classified as *S. granarius* in this tree, but it is notable that the two other individuals from the same locality are classified as *S. coronatus* based on their nuclear genotype (see below), and they came from an area that Yannic et al. [14] mapped as occupied by *S. coronatus*.

In the phylogenetic network based on *CytB* haplotypes, again, *S. granarius* haplotypes from Portugal and northwestern Spain was clearly positioned as the lineage closest to the outgroup, and the other *S. granarius* haplotypes were considerably distant on the network as part of the cluster of *S. araneus* haplotypes (Figure 2B). As also evident in Figure 2A, there was much more variation in *S. coronatus* than in *S. araneus*. This is also clear from the diversity statistics, e.g., nucleotide diversity of 0.01371 (*N* = 43) for *S. coronatus* and 0.00608 (*N* = 27) for *S. araneus* (Table 1). In the network, *S. coronatus* was positioned in an intermediate position between *S. granarius* from western Iberia and *S. araneus* together with *S. granarius* from the Central System mountain range (Figure 2B).

**Figure 3:**
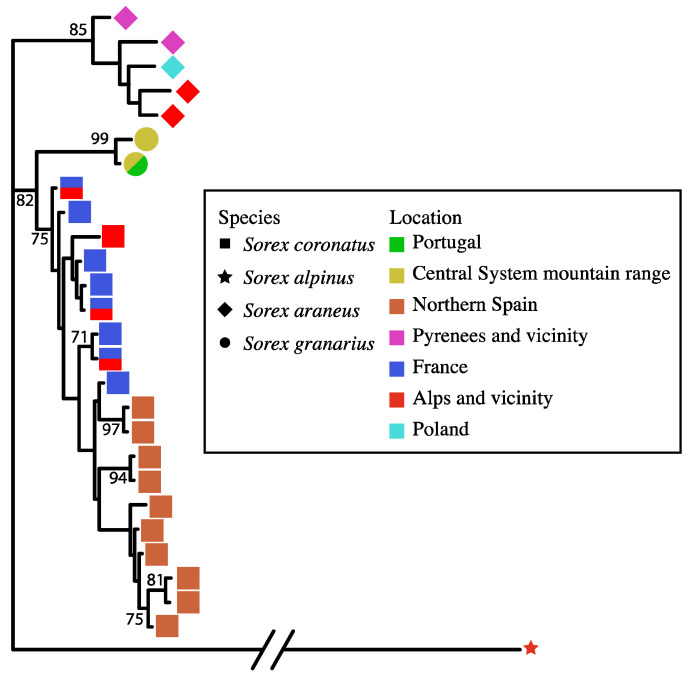
Maximum likelihood phylogenetic tree for members of the *S. araneus* complex from southwestern Europe and Poland, based on concatenated nuclear DNA sequences (1967 bp total) derived from *ApoB*, *BRCA1*, *DBY7*, and *ZFX1* sequences. Only haplotypes are shown, with geographic location and species indicated by symbols. *S. alpinus* is the outgroup. Bootstrap support over 1000 replicates is indicated on major internal nodes (only values of 70% or greater).

Altogether, 38 individuals were genotyped at all four nuclear loci (*DBY7*, *ZFX1*, *ApoB*, *BRCA1*), and Figure 3 shows their relationships in a concatenated phylogeny based on a total of 1967 bp. *S. araneus*, *S. coronatus*, and *S. granarius* all formed well-supported clades. Although not strongly supported, there are indications of differentiation in *S. coronatus* between northern Spain and elsewhere in their range (Figure 3).

**Figure 4:**
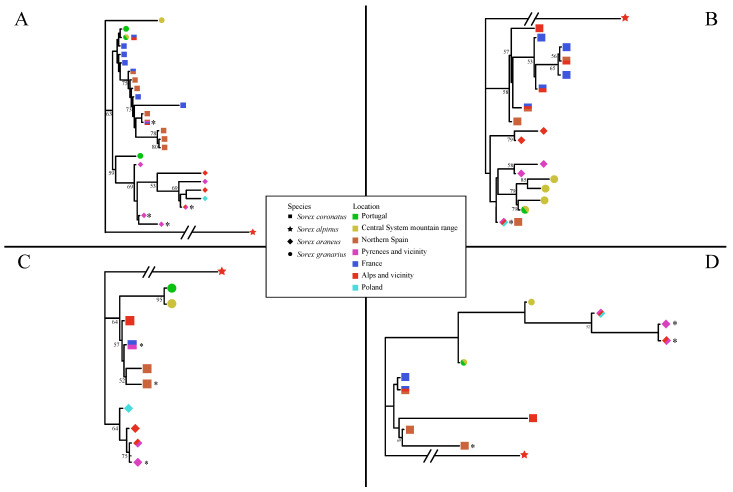
Maximum likelihood phylogenetic trees for sequences of the autosomal genes *ApoB* (**A**) and *BRCA1* (**B**), the Y-linked marker *DBY7* (**C**), and the X-linked gene *ZFX1* (**D**) of members of the *S. araneus* complex from southwestern Europe and Poland. Only haplotypes are shown, with geographic location and species indicated by symbols. *S. alpinus* is the outgroup. Asterisks indicate the haplotypes of individuals from geographic regions where two species occur. Their species designation is justified in the text. Bootstrap support over 1000 replicates is indicated on major internal nodes (only values of 50% or greater).

The ML trees for the two autosomal markers—*ApoB* and *BRCA1*—are depicted in Figure 4A,B, respectively. Contrary to what was found for *CytB*, neither marker showed a well-supported phylogenetic relationship between *S. granarius* and any *S. araneus.* For *BRCA1*, the *S. granarius* from the Central System formed a well-supported clade together with *S. granarius* from Portugal, distinct from all other *Sorex* species. This *S. granarius* clade is within a clade that is otherwise dominated by *S. araneus*, but any phylogenetic association between *S. granarius* and *S. araneus* has very low bootstrap support. For *ApoB*, the *S. granarius* are more scattered in the tree, but one of the Central System haplotypes is identical to a haplotype found in *S. granarius* in Portugal, and all of the *S. araneus* were grouped together in a clade with bootstrap support just below the 70% threshold.

For the Y-linked marker *DBY7*, the ML tree showed a similar result to the autosomal markers, with the *S. granarius* from the Central System and Portugal forming a highly supported monophyletic group, and all *S. araneus* also forming a clade (though not strongly supported) elsewhere in the tree (Figure 4C). For the X-linked marker *ZFX1*, *S. araneus* was not phylogenetically discrete from *S. granarius*, although the support for the all of the deeper nodes within the tree was very low (Figure 4D).

The two individuals typed at nuclear markers from the contact area of *S. coronatus* and *S. granarius* in northwestern Spain were classified as *S. coronatus*, in contrast with the findings for the mitochondrial marker (see above; Supplementary Table S2). They were typed at two nuclear markers each (*DBY7* and *ZFX1*, and *ApoB* and *ZFX1*, respectively; Supplementary Table S2), and the positioning of the shrews in each case was clearly within the expectations for *S. coronatus* and distinct from those for *S. granarius* (Figure 4). For the contact area of *S. coronatus* and *S. araneus* in the Pyrenees, all five individuals were typed at *ApoB* (Supplementary Table S2) and were present in the same species clade or grouping as expected on the basis of their *CytB* haplotype (Supplementary Table S2; Figure 2 and Figure 4A). There was one individual without a *CytB* haplotype whose identity as *S. araneus* was solely based on the *ApoB* result (SM.2182; Supplementary Table S2; Figure 4A). Other nuclear markers supported the identification of individuals from this contact area. However, there was an unexpected aspect to the results for *BRCA1* when considering a wider distribution area (Figure 4B). Individuals otherwise classified as *S. coronatus* from northern Spain (in Cantabria (SM.CA.0340, 1187), Burgos (SM.BU.1193), and Picos de Europa (SM.AS.0323, SM.2175)) all had the same *BRCA1* haplotype as *S. araneus* from within the contact area between *S. coronatus* and *S. araneus* in the Pyrenees (SM.2178; IZEA 4354, 4355, 4357, 4359) (Supplementary Table S2; [14]). Phylogenetically, the *BRCA1* haplotype was clearly *S. araneus*.

Regarding the relationships of *S. araneus/coronatus/granarius*, in the *CytB* tree, *S. araneus* and *S. coronatus* were most closely related, while the network put the west Iberian *S. granarius* and the *S. coronatus* as most closely related. *S. coronatus* and *S. granarius* were also the most closely related species pair for *ApoB* and *DBY7.* For *BRCA1* and *ZFX1*, *S. araneus* and *S. granarius* appeared most closely related, but with very low bootstrap support in each case. In the concatenated tree for all four nuclear loci, *S. coronatus* and *S. granarius* were most closely related, with strong bootstrap support (Figure 3).

## 4. Discussion

Yannic et al. [13,14] inferred that the *S. granarius* from the Central System mountain range of Spain had a mitochondrial genome derived from *S. araneus*—specifically, from a population currently represented by *S. araneus* in the Pyrenees (i.e., the Carlit chromosomal race). They made this inference of mitonuclear discordance based on the very close similarity of the *S. granarius CytB* sequence to that of the Pyrenean *S. araneus*. In contrast, a range of nuclear markers showed *S. granarius* and *S. araneus* to be phylogenetically well distinct. Thus, they presumed that *S. granarius* ancestrally had a distinctive nuclear and mitochondrial genome, and that it was only upon hybridisation with *S. araneus* that they lost their own mitochondrial genome and gained that of *S. araneus*. However, the existence of a distinctive “original” *S. granarius* mitochondrial genome was only conjecture—they did not demonstrate it.

Here, we show that there is a distinctive mitochondrial genome that can be ascribed to *S. granarius*. This is demonstrated by *CytB* sequences of eight individuals from seven localities widely spaced over Portugal and northwestern Spain (Supplementary Table S2; Figure 1). Four of these individuals from four localities have haplotypes at 2–4 nuclear loci that are phylogenetically close to those of *S. granarius* from the Central System. This suggests that *S. granarius* has both a distinctive nuclear and mitochondrial genome that is found in these four individuals. The other four individuals from western Iberia with *CytB* sequences of the *S. granarius* type did not have any nuclear data.

Thus, our results confirm Yannic et al.’s [13,14] inference that *S. granarius* “captured” the mitochondrial genome of *S. araneus*, with the original mitochondrial genome of *S. granarius* (that we described in western Iberia) being replaced by that of *S. araneus* in those nine individuals from three localities in the Central System mountain range of Spain described by Yannic et al. [14].

How might this mitochondrial capture have occurred? It must have involved hybridisation between *S. granarius* and *S. araneus* when one or both species had a wider distribution and were in contact, since currently they are allopatric (Figure 1). The *S. granarius* with the captured mitochondrial genome are found in the Central System of Spain, far from the current population of *S. araneus* with the closely similar mitochondrial genome in the Pyrenees. It seems unlikely that the introgression event occurred during the Holocene—more likely the Late Glacial, as detailed below. In a European context, all *Sorex* species are found in places with relatively cool, damp conditions [15]. Thus, in Southern Europe they occur largely in mountainous areas; the exception is the coast around northern and northwestern Iberia occupied by *S. coronatus* and *S. granarius* (Figure 1). In an Iberian context, this is a relatively cool, wet area. *Sorex* do not occur in the hot, dry conditions of central and southern lowland Iberia. Therefore, it can be presumed that the *S. granarius* in the Central System of Spain have long been separated from the *S. araneus* in the Pyrenees. It appears most likely that the two species were in contact during the Last Glaciation, when there were cooler, damper conditions in Iberia [25]. Given its current restriction there, it can be suggested that *S. granarius* was localised in Iberia throughout the Last Glaciation. *S. araneus* is believed to have had a glacial refugium in southeastern Europe and spread widely eastwards, westwards, and northwards in the warmer conditions after the Last Glacial Maximum (LGM) [26], and presumably reached southwestern Europe at that time. It is difficult to be exactly sure of the ranges of *S. araneus*, *S. coronatus*, and *S. granarius* during the Late Glacial period from the end of the LGM until the beginning of the Holocene—comprising the time interval of approximately 20–10,000 years ago, incorporating the Bølling–Allerød warm period followed by the Younger Dryas cold period [25]. Both climatic conditions and their competitive interactions, including competitive exclusion, would have been important [27,28]. *S. araneus* and *S. granarius* could easily have had different ranges than at present in Iberia and come into contact, creating the opportunity for hybridisation and introgression. The current distributions of the three species—with *S. araneus* and *S. granarius* not only disjunct, but with the Carlit race of *S. araneus* in the Pyrenees completely surrounded by *S. coronatus* (Figure 1)—indicate a dynamism of range changes during the Bølling–Allerød, Younger Dryas, and Early Holocene. Other examples of strange disjunct distributions deriving from range changes at this time can be seen in phylogeographic studies of other species, e.g., the distribution of the Carpathian and Eastern mitochondrial clades in the bank vole *Clethrionomys glareolus* in Central, Northern, and Western Europe [29]. Diversity statistics in Table 1 indicate that various groupings of the shrews analysed in our study have not been through extreme population bottlenecks but have undergone expansion relatively recently. The high diversity values are not surprising given that the specimens sampled usually come from wide geographical areas.

Clues as to how *S. araneus* and *S. granarius* could have come into contact in Iberia come from their current distributions and that of *S. coronatus* (Figure 1). The relative climatic tolerance of *S. araneus* and *S. coronatus* is particularly clear. Thus, where the two species are in parapatry in southwestern Europe, *S. araneus* occurs in the cooler, damper, higher-altitude areas (i.e., the Pyrenees, Massif Central). The greater tolerance of cooler, wetter conditions by *S. araneus* over *S. coronatus*—and vice versa for warmer, drier conditions—is even evident at a microgeographic level, based on studies in Switzerland [27]. Thus, through competitive exclusion, it can be conjectured that once *S. araneus* had spread to southwestern Europe after the LGM, it may have had a wider range than *S. coronatus* in northern Iberia during the Younger Dryas cold phase. This could have been the time that it came into contact with *S. granarius*, allowing capture of the *S. araneus* mitochondrial genome by *S. granarius*. This is because *S. granarius* likely occurred in lowland central Iberia at that time—rather than being restricted to the mountains, as at present—because of the overall cooler conditions. Hence, *S. araneus* expanding southwards and *S. granarius* expanding northwards could have met. At the end of the Younger Dryas, the warmer conditions would presumably have caused extinction of *Sorex* from lowland central Iberia, with *S. granarius* (with an *S. araneus* mitochondrial genome) retreating to the Central System mountain range (Figure 1). The warmer conditions of the Early Holocene apparently favoured the spread of *S. coronatus*, at the expense of *S. araneus* [30]. *S. coronatus* came to occupy almost the whole of northern Spain, France, and nearby areas (Figure 1). The spread to northern France must have occurred after 8000 years ago, because *S. coronatus* did not manage to get across the land bridge that connected to Britain until this time [30]. However, the spread must have been before 6000 years ago, because *S. coronatus* did manage to spread to the Channel Isles, which were connected to northern France until this time [31]. It can be supposed that it was at the time of the spread of *S. coronatus* that *S. araneus* became restricted to the high-altitude populations in the Pyrenees and Massif Central [30]. The *S. araneus* population in the Pyrenees may be considered to be the remnant population of that which came into contact with *S. granarius.*

There have been various suggestions as to how mitochondrial capture could be a neutral process and merely reflect the differing population dynamics of the two interacting populations (e.g., [32]). While such a neutral process may explain the mitochondrial capture of the *S. araneus* mitochondrial genome by *S. granarius* in Iberia, there is actually no a priori expectation of vastly different population size or spread between *S. araneus* and *S. granarius*. However, it is striking that in a number of mammals in Europe—e.g., *Clethrionomys* in Fennoscandia and the Urals [29], and *Lepus* in Iberia [33]—relatively warm-tolerant species have captured the mitochondrial genomes of relatively cold-tolerant species. *Sorex* in Iberia follow a similar pattern and, together with the other examples of mitochondrial introgression from a cold-tolerant to a warm-tolerant species, may indicate that the captured mitochondrial genome provides selectively favourable traits [5]. The mitochondrial genome is related to energy production, so it may be expected that a cold-adapted species would have a cold-adapted mitochondrial genome. Clearly, further bioinformatics studies of the whole mitochondrial genome and follow-up physiological studies would be necessary to confirm this suggestion. It is also worth noting that an additional case of mitochondrial capture has been described in Iberia involving wall lizards [34], so it would also be worth thinking about whether there is something specific about the conditions in Iberia that make it more likely for mitochondrial capture to happen there.

That *S. granarius* and *S. araneus* were able to hybridise is striking because every species within the *S. araneus* complex has a different karyotype, and karyotypic difference is believed to be important in promoting reproductive isolation in the group [35]. The Carlit chromosomal race of *S. araneus* has one of the simplest, least-derived karyotypes of any chromosomal race in the species [11], and this may increase the chance of successful hybridisation. The Carlit chromosomal race of *S. araneus* (2*n* = 26–29) differs from *S. granarius* (2*n* = 36–37) due to the occurrence of Robertsonian (Rb) fusions—with 4–5 metacentric chromosomes found in *S. araneus* that are present as acrocentrics in *S. granarius* [11]. In hybrids, the Rb metacentrics from *S. araneus* will, in each case, pair and recombine with—and segregate from—two homologous acrocentrics from *S. granarius*, forming trivalent configurations at meiosis I [36]. A hybrid with 4–5 of such meiotic trivalents would not be expected to suffer particularly high levels of infertility on chromosomal grounds alone [36,37]. However, given that *S. granarius* and *S. araneus* have been geographically isolated from one another and accumulated genic differences, hybrids between the species may suffer an unfitness deriving from both genic and chromosomal factors. There is a precedent in the *Sorex* shrews in Switzerland, where naturally occurring hybrids between *Sorex antinorii* and *S. araneus* have an unfitness considerably greater than expected from chromosomal difference alone [38]. However, in that case, the chromosomal difference is substantially more than between the Carlit chromosomal race of *S. araneus* and *S. granarius* and, even then, reproductive isolation is not absolute, allowing for a degree of introgression [39]. Therefore, when *S. araneus* and *S. granarius* came into contact, it is reasonable to expect that they would have been able to mate with one another, and that the hybrids—even if unfit—may have produced occasional offspring, thereby permitting genetic introgression, including mitochondrial capture.

What about the current contacts between *Sorex* species in Iberia? Based on the published distribution map (reproduced in Figure 1), *S. coronatus* and *S. araneus* are currently sympatric or parapatric in the Pyrenees, while *S. coronatus* and *S. granarius* are geographically close and possibly in contact in northwestern Spain. On chromosomal grounds, knowing that there can be some successful hybridisation and introgression between *S. araneus* and *S. granarius*, as well as between *S. antinorii* and *S. araneus*, it is a possibility that there may be hybridisation and introgression between *S. coronatus* and *S. araneus*, as well as between *S. coronatus* and *S. granarius.* This is particularly the case for *S. coronatus* (2*n* = 22–23) and *S. granarius* (2*n* = 36–37), with seven metacentrics found in *S. coronatus* that are present as acrocentrics in *S. granarius* [11], together with three additional non-Rb chromosomal rearrangements distinguishing the two species [40]. This is a greater difference than that between *S. granarius* and the Carlit chromosomal race of *S. araneus*, but not massively so. For *S. coronatus* and the Carlit chromosomal race of *S. araneus*, the chromosomal difference is very substantial [11,40].

Indeed, our genetic data do suggest that there may have been hybridisation and introgression between all pairs of *Sorex* species in Iberia. Thus, in Illano in northwestern Spain, two of the four shrews only have data for *CytB* and are categorised as *S. granarius*, while two only have data for nuclear markers and are categorised as *S. coronatus* (Supplementary Table S2; Figure 1). If all four individuals represent the norm for this population, this could be an example of cytonuclear discordance, i.e., an *S. coronatus* nuclear background with an *S. granarius* mitochondrial genome. More data are needed to be certain of this. For *BRCA1*, it is striking that exactly the same haplotype as found in the Carlit chromosomal race of *S. araneus*, located in the Pyrenees, occurs in *S. coronatus* in northern Spain going west from the Pyrenees (Figure 1 and Figure 4B). Five out of eighteen individuals from this region have the *S. araneus* haplotype for *BRCA1*, while the others have haplotypes that are phylogenetically within *S. coronatus*. All five individuals with the *S. araneus* haplotype for *BRCA1* are characterised as *S. coronatus* based on 3–4 additional genetic markers and are within the previously described distribution area of *S. coronatus* (Figure 1). It is difficult to suggest this as anything other than an introgression event, i.e., that *S. coronatus* and *S. araneus* hybridised and, with subsequent backcrossing, there are individuals with largely *S. coronatus* genomes but with an *S. araneus* allele at *BRCA1*. The fact that the *BRCA1* haplotype in *S. coronatus* is identical to the *BRCA1* haplotype in *S. araneus* suggests that the introgression event is recent (maybe during the Late Holocene) and presumably occurred in the vicinity of the Pyrenees, with the *S. araneus* allele spreading westward thereafter. To fully understand the complexity of the introgression events involving *S. coronatus*, there is a need to sample the species more intensively across Spain—and likewise with the neighbouring populations of *S. araneus* and *S. granarius.* Clearly, the Iberian *Sorex* would be best studied using genomics, to be able to determine exactly which parts of the genome have introgressed. Such an approach has been adopted successfully in hares occurring in the Iberian Peninsula and elsewhere [41].

## 5. Conclusions

Based on their previous studies, Yannic et al. [13,14] proposed a mitochondrial capture involving *Sorex* species in Iberia. Their inference was based on the substantial nuclear divergence and virtual identity in mitochondrial *CytB* sequences between *S. granarius* and *S. araneus*, with the hypothesis that *S. granarius* captured the *S. araneus* mitochondrial genome. Yannic et al. [13,14] had only genotyped *S. granarius* from a small part of their range. In our new sampling of *S. granarius*, we were able to find individuals that had both nuclear and mitochondrial genomes distinct from those of *S. araneus*. Thus, we believe that we have found the ancestral mitochondrial genome of *S. granarius*—replaced by the *S. araneus* mitochondrial genome in populations from the Central System of Spain—providing the “missing piece” in the hypothesis of Yannic et al. [13,14]. Furthermore, we provide a biogeographical scenario for the mitochondrial capture, and place the capture in the context of karyotypic differences between the species. We also discuss whether natural selection favoured *S. granarius* with the captured mitochondrial genome, but further studies are needed to address this more fully. Finally, our data suggest that there have been other introgression events among the three species of *Sorex* in Iberia.

## Data Availability

All the sequences have been deposited in the European Nucleotide Archive (ENA) at EMBL-EBI under accession number PRJEB57779 (https://www.ebi.ac.uk/ena/browser/view/PRJEB57779).

## Supplementary Materials

The following supporting information can be downloaded at: https://www.mdpi.com/article/10.3390/genes13122228/s1, Table S1: Details of the loci, primers, and annealing temperatures used in this study [42,43,44,45,46]. Table S2: All Sorex from Portugal, Spain, France, and Andorra that were genetically typed in this study for various combinations of the mitochondrial locus *CytB* and the nuclear loci *ApoB*, *BRCA1*, *DBY7*, and *ZFX1*. They were classified as *S. araneus*, *S. coronatus*, or *S. granarius* based on geography [14] or genotype at nuclear or mitochondrial DNA loci (see text).
