# Supplementary material for: Characterising Mitochondrial Capture in an Iberian Shrew
Source: Genes (Basel). 2022 Nov 27;13(12):2228. doi: 10.3390/genes13122228 (PMC9777731; doi:10.3390/genes13122228)
Supplement: Supplementary file 1 [file genes-13-02228-s001.zip › genes-1986593-supplementary.pdf]

Table S1. Details of the loci, primers, and annealing temperatures used in this study.

Locus	Size (bp)	Forward primer	Reverse primer	Reference	Tm (°C)
CytB	1003	L14723<br>ACCAATGACATGAAAAATCATCGTT	H15915-S<br>TCTCCATTTCTGGTTTACAAGAC	[42]	55
		L14727-SP<br>GACAGGAAAAATCATCGTTG	H15915-SP<br>TTCATTACTGGTTTACAAGAC	[43]	55
DBY7	504	DBY7 F<br>GGTCCAGGAGARGCTTTGAA	DBY7 R<br>CAGCCAATTCTCTTGTTGGG	[44]	TD 65-55
ApoB	499	APOB-F<br>GCAATCATTTGACTTAAGTG	APOB-R<br>GAGCAACAATATCTGATTGG	[45]	60
BRCA1	774	B1F<br>TGAGAACAGCACTTTATTACTCAC	B1R<br>ATTCTAGTTCCATATTGCTTATACTG	[46]	60
ZFX1	190	ZFX1SA-F<br>GGTTAAAAGATGGGGCTGGAGA	ZFX1SA-R<br>GCATGAGCGGTCAAGTTCTGTT	This study	64

bp: base pairs; Tm: primer melting temperature; TD: touchdown PCR

Table S2. All *Sorex* from Portugal, Spain, France, and Andorra that were genetically typed in this study for various combinations of the mitochondrial locus *CytB* and nuclear loci *ApoB*, *BRCA1*, *DBY7*, and *ZFX1*. They were classified as *S. araneus*, *S. coronatus*, or *S. granarius* based on geography [14] or genotype at nuclear or mitochondrial DNA loci (see text).

Code	Geographical species	nuDNA species	mtDNA species	Locality	Country	*CytB*	*ApoB*	*BRCA1*	*DBY7*	*ZFX1*
SM.AS.0323	*coronatus*	*coronatus*	*coronatus*	Picos de Europa	Spain	X	X	X		X
SM.CA.0340	*coronatus*	*coronatus*	*coronatus*	Cantabria	Spain	X	X	X	X	X
SM.AS.0448	*coronatus/granarius*	-	*granarius*	Illano	Spain	X				
SM.AS.0449	*coronatus/granarius*	*coronatus*	-	Illano	Spain				X	X
SM.AS.0451	*coronatus/granarius*	-	*granarius*	Illano	Spain	X				
SM.AS.0452	*coronatus/granarius*	*coronatus*	-	Illano	Spain		X			X
SM.AS.1180	*coronatus*	*coronatus*	*coronatus*	Picos de Europa	Spain	X	X	X	X	X
SM.AS.1181	*coronatus*	*coronatus*	*coronatus*	Picos de Europa	Spain	X	X	X	X	X
SM.AS.1182	*coronatus*	*coronatus*	*coronatus*	Picos de Europa	Spain	X	X	X	X	X
SM.AS.1183	*coronatus*	*coronatus*	-	Picos de Europa	Spain		X	X	X	X
SM.AS.1185	*coronatus*	*coronatus*	*coronatus*	Picos de Europa	Spain	X				X
SM.CA.1187	*coronatus*	*coronatus*	*coronatus*	Cantabria	Spain	X	X	X	X	X
SM.CA.1188	*coronatus*	*coronatus*	*coronatus*	Cantabria	Spain	X	X	X	X	X
SM.CA.1189	*coronatus*	*coronatus*	*coronatus*	Cantabria	Spain	X	X		X	X
SM.PB.1190	*coronatus*	*coronatus*	*coronatus*	Bizcaia	Spain	X	X	X		X
SM.PB.1191	*coronatus*	*coronatus*	*coronatus*	Bizcaia	Spain	X	X	X	X	X
SM.PB.1192	*coronatus*	*coronatus*	*coronatus*	Bizcaia	Spain	X	X	X		
SM.BU.1193	*coronatus*	*coronatus*	-	Burgos	Spain		X	X	X	X
SM.BU.1194	*coronatus*	*coronatus*	-	Burgos	Spain		X	X	X	X
SM.BU.1195	*coronatus*	*coronatus*	*coronatus*	Burgos	Spain	X	X	X		X
SM.BU.1196	*coronatus*	*coronatus*	*coronatus*	Burgos	Spain	X	X	X		X
SM.BU.1197	*coronatus*	*coronatus*	*coronatus*	Burgos	Spain	X	X	X	X	X
SM.1344	*granarius*	-	*granarius*	Bertiandos	Portugal	X				
SM.1990	*granarius*	*granarius*	*granarius*	Parque Nac. Peneda Geres	Portugal	X	X	X	X	X
SM.2175	*coronatus*	*coronatus*	*coronatus*	Picos de Europa	Spain	X	X	X		X
SM.2176	*coronatus*	*coronatus*	*coronatus*	Burgos	Spain	X	X	X		X
SM.2178[1]	*araneus/coronatus*	*araneus*	*araneus*	Pyrenees	Spain	X	X	X		X
SM.2179[2]	*araneus/coronatus*	*araneus*	*araneus*	Pyrenees	Spain	X	X		X	X

SM.2182[3]	*araneus/coronatus*	*araneus*	-	Vall d'Aran	Spain		X			
SM.2183[4]	*araneus/coronatus*	*coronatus*	*coronatus*	Eugui	Spain	X	X		X	
SM.2184[5]	*araneus/coronatus*	*araneus*	*araneus*	Tarter Pas Casa	Andorra	X	X			X
SM.2196	*coronatus*	-	*coronatus*	Baie de l'Aiguillon	France	X				
SM.2199	*coronatus*	*coronatus*	*coronatus*	Abbeville	France	X	X	X	X	X
SM.2200	*coronatus*	*coronatus*	-	Abbeville	France			X	X	X
SM.2201	*coronatus*	*coronatus*	*coronatus*	Calais	France	X	X	X	X	X
SM.2202	*coronatus*	*coronatus*	*coronatus*	Calais	France	X	X	X	X	X
SM.2203	*coronatus*	*coronatus*	*coronatus*	Calais	France	X	X	X		X
SM.2204	*coronatus*	*coronatus*	*coronatus*	Broualan	France	X	X	X	X	X
SM.2206	*coronatus*	*coronatus*	*coronatus*	Mas d'Artige	France	X	X	X	X	
SM.2207	*coronatus*	*coronatus*	*coronatus*	St Setiers	France	X	X	X	X	X
SM.2208	*coronatus*	*coronatus*	*coronatus*	St Setiers	France	X	X	X	X	X
SM.2209	*coronatus*	*coronatus*	*coronatus*	St Setiers	France	X	X	X	X	X
SM.2210	*coronatus*	*coronatus*	*coronatus*	St Setiers	France	X	X	X	X	X
SM.2211	*coronatus*	*coronatus*	*coronatus*	Troarn	France	X	X	X		X
SM.2212	*coronatus*	*coronatus*	*coronatus*	Broualan	France	X	X	X	X	X
SM.2213	*coronatus*	*coronatus*	*coronatus*	Broualan	France	X	X		X	
SM.2214	*coronatus*	*coronatus*	*coronatus*	Beauvoir	France	X	X	X	X	X
SM.2215	*coronatus*	*coronatus*	-	Beauvoir	France		X	X	X	
SM.2216	*coronatus*	*coronatus*	*coronatus*	Beauvoir	France	X	X	X	X	X
SM.2217	*coronatus*	*coronatus*	*coronatus*	Mas d'Artige	France	X	X	X		
SM.2218	*coronatus*	*coronatus*	*coronatus*	Mas d'Artige	France	X	X	X	X	
SM.2219	*coronatus*	*coronatus*	*coronatus*	Mas d'Artige	France	X	X	X		X
SM.2222	*granarius*	*granarius*	*granarius*	Samarra	Portugal	X	X			X
SM.2224	*granarius*	*granarius*	*granarius*	Serra da Estrela	Portugal	X	X			X
SM.2225	*granarius*	*granarius*	*granarius*	Sintra	Portugal	X	X	X		X
SM.2228	*coronatus*	*coronatus*	-	Calais	France				X	X
SM.2229	*coronatus*	*coronatus*	-	Calais	France					X
SM.2230	*coronatus*	*coronatus*	-	Mt St Michel	France		X			X
SM.2231	*coronatus*	*coronatus*	*coronatus*	Mt St Michel	France	X	X			X
SM.2232	*coronatus*	*coronatus*	-	Calais	France		X			X

SM.2433	*granarius*	-	*granarius*	Vila-Chã	Portugal	X				

[1]Granollers Museum code: 96116112
[2]Granollers Museum code: 96041911
[3]Granollers Museum code: 98063001
[4]Granollers Museum code: 96091009
[5]Granollers Museum code: P1/AND1
